# Curcumin ameliorates atrophy of seminal vesicle via reduction of oxidative stress in castrated mice

**DOI:** 10.7717/peerj.7192

**Published:** 2019-07-05

**Authors:** Rui Li, Hao Li, Ke Rao, Kang Liu, Yan Zhang, Xiaming Liu, Tao Wang, Shaogang Wang, Zhuo Liu, Jihong Liu

**Affiliations:** Department of Urology, Tongji Hospital, Tongji Medical College, Huazhong University of Science and Technology, Wuhan, People’s Republic of China; Institute of Urology, Tongji Hospital, Tongji Medical College, Huazhong University of Science and Technology, Wuhan, People’s Republic of China

**Keywords:** Castration, Curcumin, Oxidative stress, Seminal vesicle

## Abstract

**Background:**

The growth and function of seminal vesicle are dependent on androgen. This study was conducted to investigate the role of oxidative stress in castration-induced seminal vesicle atrophy and to explore the effects of curcumin, an antioxidant extracted from rhizome of turmeric, on seminal vesicle of castrated mice.

**Methods:**

C57BL/6J mice were randomly divided into three groups: control, castration, and castration with curcumin (*n* = 10 for each group). After surgical castration, mice in the curcumin treatment group received intragastric administration of curcumin at 100 mg/kg body weight for 4 weeks, whereas mice in the other two groups were treated with olive oil. After that, the body weight, seminal vesicle weight and serum testosterone of mice were measured. Apoptosis and oxidative stress levels in seminal vesicle were also determined.

**Results:**

After castration, both the weight and size of seminal vesicle decreased dramatically. The expression of three NADPH oxidase (NOX) subtypes: NOX1, NOX2 and NOX4, increased in seminal vesicle of castrated mice, resulting in high level oxidative stress. The ratio of Bax to Bcl-2 was also elevated after castration, accompanied by enhanced caspase3 activity. Additionally, castration increased the number of apoptotic cells in seminal vesicle. Curcumin treatment could inhibit the expression of NOX1, NOX2 and NOX4, decreasing oxidative stress and apoptosis. The atrophy of seminal vesicle caused by castration was ameliorated by curcumin.

**Conclusion:**

Castration could cause atrophy of seminal vesicle probably via inducing oxidative stress. Curcumin treatment could reduce the oxidative stress in seminal vesicle by decreasing the expression of NOX1, NOX2 and NOX4, thereby ameliorating apoptosis and atrophy of seminal vesicle. Oxidative stress might play a role in castration-induced seminal vesicle atrophy.

## Introduction

Infertility affects nearly one in five couples worldwide, with 30% of infertility cases caused by male factors and 20% attributed to a combination of female and male factors (Hu et al., 2016). In addition to the impaired sperm quality, a low volume of seminal plasma may also lead to infertility (Roberts & Jarvi, 2009). Seminal plasma plays an important role in the maturation, transport, and storage of spermatozoa (Tian et al., 2016; Piehl et al., 2013). Seminal vesicle fluid is a major component of seminal plasma. 60–70% of seminal plasma is produced from seminal vesicle and 20–30% from the prostate (Pei et al., 2013).

The growth and function of seminal vesicle are highly dependent on androgens (Ilbey et al., 2009). A previous study showed that patients with androgen deficiency had a smaller volume of semen compared with normal men (Aksglaede et al., 2009). Treatment with androgens could increase the secretion of seminal vesicle in men (Gonzales, 1994). Animal studies indicated that androgen supplement increased both the weight and secretory activity of seminal vesicle in rats (Almenara et al., 2001; Tao, Lei & Rao, 1998; Zanato et al., 1994). However, whether oxidative stress plays a role in castration-induced seminal vesicle atrophy is not very clear at present.

Reactive oxygen species (ROS) are predominantly generated from mitochondrial respiration and nicotinamide adenine dinucleotide phosphate (NADPH) oxidase (NOX) (Hu et al., 2018).**** Excess production of ROS can result in oxidative stress, damaging DNA and proteins in cells (Martindale & Holbrook, 2002). Studies showed that castration caused a dramatic increase of ROS in some organs of the male reproductive system such as prostate and corpus cavernosum, leading to cell apoptosis (Li et al., 2016; Tam et al., 2003). In addition, castration could also enhance oxidative stress in the bladder wall and urethral sphincter of rats (Ludwig et al., 2011; Kracochansky et al., 2012). We thereby speculated that androgen deficiency may cause the atrophy of seminal vesicle via an elevation of the ROS level.

Curcumin is a curcuminoid from the rhizome of turmeric. It has long been used to treat diseases in traditional Chinese medicine. Many studies indicate that curcumin possesses antioxidant, anti-inflammatory and antitumor properties (Sahoo, Roy & Chainy, 2008; Shu et al., 2009; Mathuria & Verma, 2008). It has been used as an antioxidant in many studies. For example, curcumin showed a protective effect on the oxidative injury in the testes of rats (Ilbey et al., 2009). Oxidative stress indexes such as protein carbonyl and lipid peroxide in testes were reduced to a normal level by curcumin in hyperthyroid rats. However, superoxide dismutase (SOD) was elevated after curcumin treatment (Sahoo, Roy & Chainy, 2008).

This study was conducted to investigate the role of oxidative stress in castration-induced seminal vesicle atrophy. Curcumin was used as an antioxidant to reduce oxidative stress. The effect of curcumin on oxidative stress and apoptosis in seminal vesicle of castrated mice was explored.

## Materials and Methods

### Treatment of animals

This study was approved by the Animal Care and Use Committee of Tongji Hospital, Tongji Medical College, Huazhong University of Science and Technology, Wuhan, China (TJ-A20180601). 30 male, 8-week-old specific-pathogen-free C57BL/6J mice obtained from the Laboratory Animal Center of Tongji Medical College, Huazhong University of Science and Technology, were randomly divided into three groups: control, surgical castration, and castration with curcumin (*n* =10 for each group). To perform surgical castration, mice were anesthetized by intraperitoneal injection of pentobarbital sodium (60mg/kg body weight; Sigma-Aldrich, P3761, St. Louis, MO, USA) and fixed on a warm pad in a supine position. An incision was created above the scrotum at each side and testis was squeezed out from the incision. The spermatic cord was separated and ligated, followed by the removal of the testis. Finally, the wounds were sutured and disinfected. Animals in the control group received sham operation with only scrotum incision. After these procedures, the mice were placed in a clean and warm environment to revive from anesthesia.

The second day after the operation, mice in the curcumin treatment group began to receive intragastric administration of curcumin dissolved in olive oil (Sigma-Aldrich; 08511) at 100 mg/kg body weight for 4 weeks, whereas mice in the other two groups were only treated with olive oil by gavage. The dose was chosen based on a previous study (Awad & El-Sharif, 2011). All mice were monitored carefully for three days after surgery to make sure that the wounds were not dehiscent or infected.

After 4 weeks, all mice were weighed and anesthetized. The abdomen was opened by a midline incision. Bilateral seminal vesicles were harvested and the seminal vesicle fluid was squeezed out gently using a curved tweezer. After weighing, one of the seminal vesicles was snap frozen with liquid nitrogen and stored at −80 °C, while the other was immersed in 4% paraformaldehyde for paraffin embedding. A Blood sample was collected from the abdominal vein and left to clot at room temperature for 1 h. The serum was collected and stored at −80 °C.

### Measurement of serum testosterone

Serum T levels were measured with an Elisa Kit (ab108666; Abcam, Cambridge, UK). Briefly, duplicate 25 ul of the standards, control and samples were added to the respective wells of the ELISA plate, followed by the addition of 100 ul of Testosterone-HRP Conjugate into each well. After 1 h of incubation at 37 °C, each well was washed three times with 300 ul of Wash Buffer. 100 ul TMB Substrate Solution was added into the wells with 15-minute incubation at 37 °C in the dark. After adding 100ul Stop Solution, the absorbance was read at 450 nm on a microplate reader (Thermo Fisher, Waltham, MA, USA). The concentration of testosterone of each sample was calculated according to the standard curve.

### Western blot

The cryopreserved seminal vesicles were ground and incubated with lysis buffer to extract the protein. 40 µg protein lysate of each sample was electrophoresed in sodium dodecyl sulfate/polyacrylamide (SDS-PAGE) gel and transferred to a polyvinylidene difluoride membrane (Millipore, Burlington, MA, USA). Then the membrane was blocked with 5% bovine serum albumin (Sigma-Aldrich; V900933) for 1 h and incubated with the primary antibodies at 4 °C overnight, including antibodies of Bax (1:500; Affinity, AF0120, Wuhan, China), Bcl-2 (1:500; Affinity, AF6139), NOX1 (1:500; Abcam, ab55831), NOX2 (1:500; Abcam, ab80508), NOX4 (1:500; Servicebio, GB11347, Wuhan, China),and *β*-actin (1:500; BM0627; Boster, Wuhan, China). After incubation with horseradish peroxidase-conjugated secondary antibodies (1:1000; 7074, 7076; CST, Danvers, MA, USA), the membranes were developed using Bio-Rad Clarity Western ECL Substrate (1705061; Bio-Rad Laboratories, Hercules, CA, USA). Protein bands were analyzed with Image J software (National Institutes of Health, Bethesda, MD, USA).

### Caspase3 activity measurement

Caspase3 activity in seminal vesicle was measured with a caspase3 activity assay kit (C1115; Beyotime, Shanghai, China). Briefly, samples were ground and incubated with lysis buffer on the ice. After centrifugation at 16,000 g, the supernatant was collected and mixed with acetyl-Asp-Glu-Val-Asp p-nitroanilide (Ac-DEVD-pNA). Caspase3 can convert Ac-DEVD-pNA to pNA, making the liquid turn yellow. Subsequently, the absorbance of the mixture was detected at 405 nm on a microplate reader (Thermo). According to the standard curve, the pNA concentration in each sample was calculated, with more pNA indicating a higher caspase3 activity. The caspase3 activity of each sample was normalized by its protein concentration (Li et al., 2018).

### Malondialdehyde (MDA) measurement

Lipid peroxidation is a major event caused by oxidative stress. MDA is an aldehydic metabolite of lipid peroxidation and can reflect the level of oxidative stress (Wang et al., 2017). The content of MDA in seminal vesicle was measured by an MDA assay kit (Beyotime, S0131). Briefly, samples were ground and incubated with lysis buffer. Then the tissue homogenate was centrifuged at 10000g for 10min to obtain the supernatant. 0.1 ml sample was mixed with 0.2 ml MDA testing reagent containing thiobarbituric acid, followed by incubation in boiling water for 15 min. After cooling, the mixture was centrifuged at 1000 g for 10 min. 0.2 ml supernatant was removed to a 96-well plate and measured at 532 nm on a microplate reader (Thermo). The level of MDA in each sample was calculated based on the standard curve and normalized by its protein concentration.

### Terminal deoxynucleotidyl transferase 2′-deoxyuridine 5′-triphosphate nick end labeling (TUNEL)

Paraffin-embedded tissues were cut into sections with a thickness of 8 µm. TUNEL staining was performed on the sections with an In Situ Cell Death Detection Kit (06432344001; Roche Applied Science, Indianapolis, IN, USA) according to the instructions. Nuclei of apoptotic cells were stained brown, while the nuclei in normal cells were blue. The number of apoptotic and total cells was counted in three fields of each sample at 400 × magnification. The apoptotic index, namely the ratio of apoptotic cells to total cells, was calculated to reflect the level of apoptosis (Liu et al., 2017).

### Immunohistochemistry

Immunohistochemistry was performed to detect the content and location of NOX1, NOX2 and NOX4 in seminal vesicle. The paraffin-embedded tissues were cut into sections. After dewaxing and dehydration, the sections were incubated with 3% hydrogen peroxide to inactivate the endogenous peroxidase, followed by antigen recovery with boiled citrate buffer. The sections were then washed with phosphate buffer saline and blocked with goat serum. Sections were incubated with antibodies against NOX1 (1:200; Abcam, ab55831), NOX2 (1:200; Abcam, ab80508), and NOX4 (1:200; Servicebio, GB11347) at 4 °C overnight. After that, sections were incubated successively with biotinylated secondary antibody, peroxidase-conjugated streptavidin, diaminobenzidine and Harris’s hematoxylin. Images were observed with a microscope (Olympus).

### Statistical analysis

The results were analyzed with GraphPad Prism version 5.0 (GraphPad Software, San Diego, CA, USA) and were presented as mean ± standard deviation (SD). Statistical analyses were performed using one-way analysis of variance (ANOVA) followed by Tukey test. Intergroup differences were considered statistically significant with *P* < 0.05.

## Results

### Body weight, seminal vesicle weight and serum testosterone level

**Figure 1:**
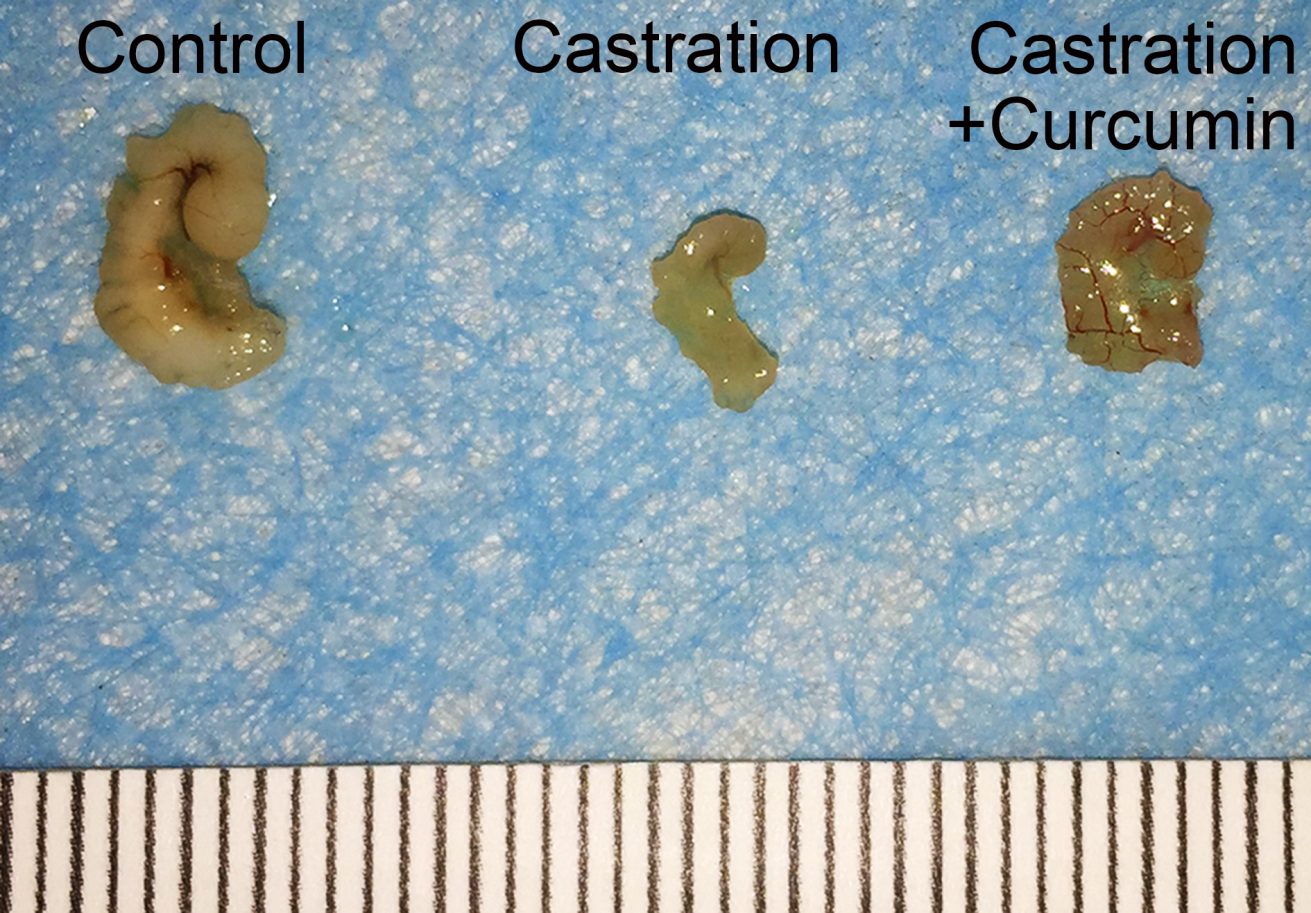
Size of seminal vesicle. Representative image of seminal vesicle from different groups.

There was no apparent difference in the body weights of mice in different groups at the beginning of the study. Castration also had no influence on the body weight of mice. On the contrary, the weight of seminal vesicle declined dramatically after castration (Table 1, 149.30 mg in control group versus 6.50 mg in castration group, *P* < 0.05). Curcumin treatment could ameliorate the weight loss of seminal vesicle to some extent, with the average seminal vesicle weight of 14.10 mg (*P* < 0.05). However, seminal vesicle weight in curcumin group was still much less than that in control group (14.10 mg versus 149.30 mg, *P* < 0.05). Furthermore, compared with the control group(3.57 pg/ml), castration caused a marked reduction in the serum testosterone level (0.53 pg/ml, *P* < 0.05). The administration of curcumin had no apparent influence on serum testosterone level in castrated mice, with an average serum testosterone level of 0.42 pg/ml. In addition to weight reduction of seminal vesicle, castration also decreased the size of seminal vesicle notably (Fig. 1). Curcumin reduced seminal vesicle atrophy in castrated mice.

**Table 1:** Body weight, seminal vesicle weight and serum testosterone level.

Group	Body weight (g)		Seminal vesicle weight (mg)	Serum testosterone (pg/mL)
	Initial	Final		
Control	20.91 ± 0.47	22.34 ± 0.53	149.30 ± 11.26	3.57 ± 0.32
Castration	21.59 ± 0.63	22.12 ± 0.50	6.50 ± 0.50^a^	0.53 ± 0.07^a^
Castration+Curcumin	21.56 ± 0.66	21.45 ± 0.43	14.10 ± 1.63^a^^,^^b^	0.42 ± 0.07^a^

**Notes.**

Data were expressed as mean ± standard deviation (SD). *n* = 10 for each group.

a *P* < 0.05 compared with control group.

b *P* < 0.05 compared with castration group.

### Apoptosis in seminal vesicle

**Figure 2:**
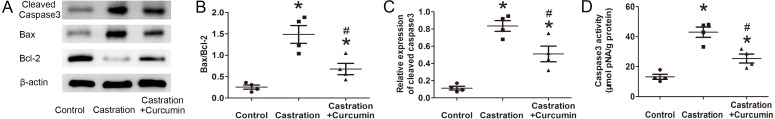
Apoptosis in seminal vesicle. (A) Representative western blot results for Bcl-2, Bax, and Cleaved caspase3. (B) Ratio of Bax to Bcl-2; *n* = 4 for each group. (C) Relative expression of Cleaved caspase3; *n* = 4 for each group. (D) Caspase3 activity detected with a kit; *n* = 4 for each group. * *P* < 0.05 compared with control group. # *P* < 0.05 compared with castration group.

Bax is a pro-apoptotic protein, whereas Bcl-2 is an anti-apoptotic protein. The ratio of Bax to Bcl-2 can be used to reflect the level of apoptosis, with a higher ratio indicating more apoptosis. Results of western blot showed that the expression of Bax in the seminal vesicle of castrated mice was higher than that in the control group. Treatment with curcumin reduced Bax expression in castrated mice. On the contrary, the content of Bcl-2 was reduced by castration and curcumin could retard the reduction (Fig. 2A). The ratio of Bax to Bcl-2 was raised by castration, but declined in the wake of curcumin treatment (*P* < 0.05, Fig. 2B). Cleaved caspase3 is the activated form of caspase3 and is considered as the executor of apoptosis. In our study, expression of cleaved caspase3 in seminal vesicle was increased after castration, along with enhanced caspase3 activity. Curcumin could reduce the expression of cleaved caspase3 and weaken caspase3 activity in castrated mice (*P* < 0.05, Figs. 2C and 2D).

**Figure 3:**
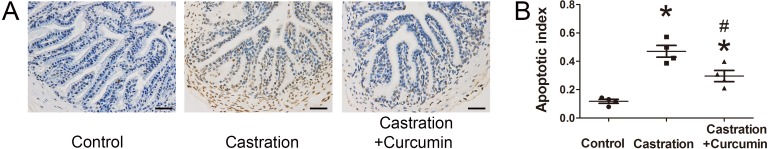
TUNEL staining and apoptotic index. (A) Representative image of terminal deoxynucleotidyl transferase 2′-deoxyuridine 5′-triphosphate nick end labeling (TUNEL) staining (400×, scale bar = 50 µm). (B) Apoptosis index detected with TUNEL staining; *n* = 4 for each group. *P < 0.05 compared with control group. # *P* < 0.05 compared with castration group.

We further performed TUNEL staining to detect apoptotic cells in seminal vesicle. Apoptotic index was calculated to indicate the percentage of apoptotic cells. Consistent with the above results, castration led to a higher apoptotic index and curcumin could lower apoptotic index in castrated mice (*P* < 0.05, Fig. 3).

### Oxidative stress level in seminal vesicle

**Figure 4:**
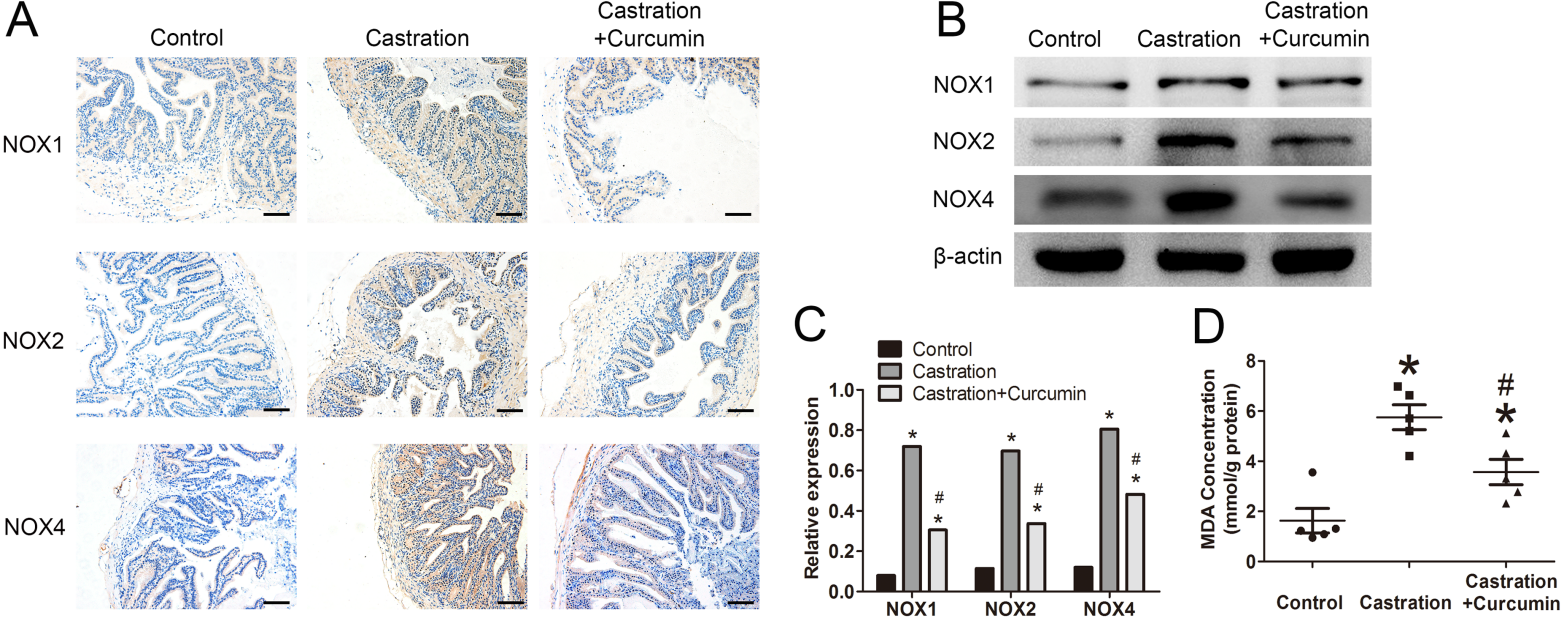
Oxidative stress in seminal vesicle. (A) Representative image of immunohistochemistry detecting NOX1, NOX2, and NOX4(200×, scale bar =100 µm). (B) Representative western blot results for NOX1, NOX2, and NOX4. (C) Relative expression of NOX1, NOX2, and NOX4 compared with *β*-actin detected with western blot; *n* = 4 for each group. (D) Malondialdehyde (MDA) concentration measured with a MDA assay kit; *n* = 4 for each group. * *P* < 0.05 compared with control group. # *P* < 0.05 compared with castration group.

We measured the expression of NOX1, NOX2 and NOX4 with immunohistochemistry and western blot. The expressions of these three proteins were all promoted by castration, whereas curcumin could inhibit the overexpression of the three proteins in seminal vesicle of castrated mice (*P* < 0.05, Figs. 4A–4C). To further determine the oxidative stress level, we measured the content of MDA in seminal vesicle. After castration, MDA concentration in seminal vesicle was increased, whereas the intervention with curcumin could reduce MDA content (*P* < 0.05, Fig. 4D).

## Discussion

Our study showed that castration caused atrophy of seminal vesicle. As an antioxidant, curcumin could reduce the oxidative stress in seminal vesicle by inhibiting the expression of NOX1, NOX2 and NOX4, thereby ameliorating apoptosis and atrophy of seminal vesicle. These findings suggest that oxidative stress might play a role in castration-induced seminal vesicle atrophy.

Seminal vesicle is a major male accessory sexual organ that secretes seminal vesicle fluid. Seminal vesicle fluid contains many substances crucial for the survival and activation of spermatozoa, including fructose, amino acids, flavins, vitamin C, some enzymes and electrolytes (Gonzales, 2001). Fructose is the main energy source for spermatozoa (Gonzales, 1994). Moreover, as the major component of seminal plasma, a deficiency in the volume of seminal vesicle fluid can directly reduce the content of seminal plasma. A sufficient volume of seminal plasma is needed to transport the spermatozoa into the uterus of female (Pei et al., 2013). After getting into the vagina, semen can form a seminal pool around the cervix, which contacts the cervical mucus column directly and makes it possible for the sperm to swim into the uterus. Sperm is not able to enter the cervix with deficient seminal plasma, thus leading to infertility. Pang, Chow & Wong (1979) found that female mice that were mated with male mice without seminal vesicles were not able to give birth to offspring.

Seminal vesicle is highly dependent on androgens for its normal structure and function. The high columnar epithelium of seminal vesicle dramatically degraded after castration, and there was a reduction in the number of luminal microvilli, secretory granules and polysomes on the endoplasmic reticulum (Epperly et al., 1984). In addition, castration may lead to impaired protein synthesis in seminal vesicle by reducing the mRNAs content of secretory proteins. Consequently, there were no sufficient proteins in the seminal plasma to support the maturation and motility of sperm (Moore, Veneziale & Wieben, 1986). Additionally, the expression of aquaporins on the glandular epithelium of seminal vesicle was decreased by castration, resulting in deficient water secretion to the seminal vesicle lumen (Tian et al., 2016). In accordance with these findings, we also found that the weight and the size of seminal vesicle were both decreased in castrated mice.

Androgen deprivation changed the expression of some oxidative stress-related genes, such as SOD and glutathione peroxidase 1 in the prostate, which were both endogenous antioxidants (Pang et al., 2002). The imbalance of redox status might be a cause of castration-induced apoptosis. A study showed that castration increased mRNA levels of NOX1, NOX2 and NOX4, along with a reduction of SOD and glutathione peroxidase 1 (Tam et al., 2003). NOX is a membrane-bound enzyme complex that can use NADPH as electron donor to convert oxygen to superoxide or *H*_2_*O*_2_. NOX family consists of multiple isoforms, in which NOX1, NOX2 and NOX4 are abundantly expressed in rodent animals (Manea et al., 2018). NOX3 is expressed only in the inner ear and is responsible for the otoliths formation (Brandes, Weissmann & Schröder, 2014). NOX is a major source of superoxide and its overexpression usually initiates oxidant anabolism pathway (Vignais, 2002). In our study, castration elevated the expression of NOX1, NOX2 and NOX4 in seminal vesicle, while curcumin could reduce NOXs expression.

It is widely accepted that oxidative stress could cause cell apoptosis and was thought to be an apoptotic stimulus (Shukla, Banerjee & Tripathi, 2018; Fu et al., 2016). Apoptotic process is under the regulation of various proteins, such as caspases and Bcl-2 family. Bcl-2 family contains pro-apoptotic proteins such as Bax, and anti-apoptotic proteins such as Bcl-2. The balance between these two groups is necessary for cell survival (Green & Kroemer, 2004). Oxidative stress could reduce the expression of Bcl-2 and increase Bax expression in multiple cells, leading to apoptosis via release of cytochrome *c* and subsequent activation of caspase3** (Li et al., 1998; Verzola et al., 2002; Kim & Kang, 2006; Zou et al., 1997). Antioxidants can recover the balance of redox, thereby reducing apoptosis (Wagener et al., 2009; Lee et al., 2007).

To investigate the role of oxidative stress in the atrophy of seminal vesicle, we treated castrated mice with curcumin, a polyphenol extracted from turmeric with antioxidant property. Our result demonstrated that curcumin could ameliorate the oxidative stress and apoptosis in seminal vesicle of castrated mice. The weight and size of seminal vesicle were all increased after intervention with curcumin. Curcumin is a widely used antioxidant. Ilbey et al. explored the effect of curcumin in cisplatin induced testicular toxicity (Ilbey et al., 2009), showing that curcumin treatment ameliorated the oxidative injury in rat testes and prevented the reduction of testicular weight. Similarly, a study by Sahoo, Roy & Chainy (2008) demonstrated that curcumin could decrease the oxidative stress in the testes of L-thyroxine-induced hyperthyroid rats by elevating SOD and catalase level and decreasing glutathione peroxidase activity. Another study found that curcumin had a protective effect on the secretion function of seminal vesicle in mice treated with metronidazole (Noorafshan & Karbalay-Doust, 2012).

As far as we know, our study is the first to investigate the role of oxidative stress in seminal vesicle atrophy caused by androgen deficiency. However, there were still some limitations in this study. First of all, the amount of seminal vesicle fluid was too little to quantify and to perform seminal plasma biochemistry. Hence the changes of each constituent in seminal vesicle fluid were not clear. This could be solved by using bigger animals such as rabbits and monkeys in future studies. Secondly, we did not investigate how castration enhanced the expression of NOXs in seminal vesicle. The underlying mechanism needs further exploration.

## Conclusions

After castration, the seminal vesicle atrophied severely, accompanied by a high level of apoptosis and oxidative stress in seminal vesicle. Curcumin treatment could ameliorate the atrophy of seminal vesicle via reducing oxidative stress. We thereby speculate that oxidative stress may play a role in castration-induced seminal vesicle atrophy.

##  Supplemental Information

10.7717/peerj.7192/supp-1 Supplemental Information 1 Raw band figures of Western blot

10.7717/peerj.7192/supp-2 Supplemental Information 2 Raw data of caspase3 activity

10.7717/peerj.7192/supp-3 Supplemental Information 3 Raw data of MDA concentration

## Additional Information and Declarations

### Data Availability

The following information was supplied regarding data availability: Raw data are available as Supplemental Files.
