# Supplementary figures and images for: Curcumin ameliorates atrophy of seminal vesicle via reduction of oxidative stress in castrated mice
Source: PeerJ. 2019 Jul 5;7:e7192. doi: 10.7717/peerj.7192 (PMC6613531; doi:10.7717/peerj.7192)

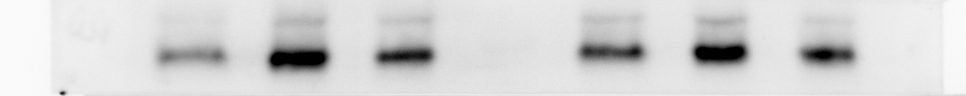

Supplement: Supplemental Information 1 [file peerj-07-7192-s001.zip › western blot/Figure2A Bax.tif]

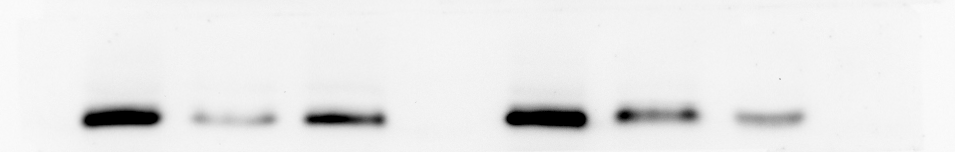

Supplement: Supplemental Information 1 [file peerj-07-7192-s001.zip › western blot/Figure2A Bcl-2.tif]

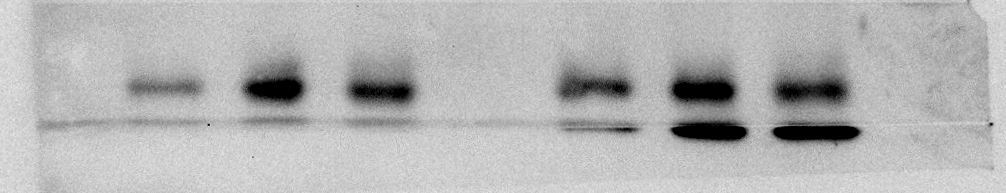

Supplement: Supplemental Information 1 [file peerj-07-7192-s001.zip › western blot/Figure2A Cleaved caspase3.tif]

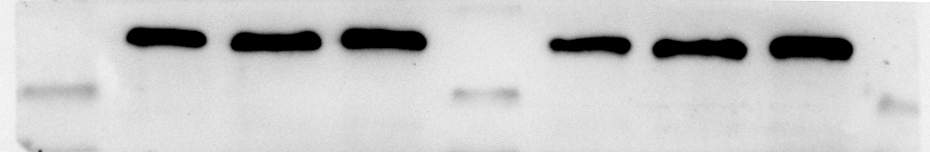

Supplement: Supplemental Information 1 [file peerj-07-7192-s001.zip › western blot/Figure2A a┬-actin.tif]

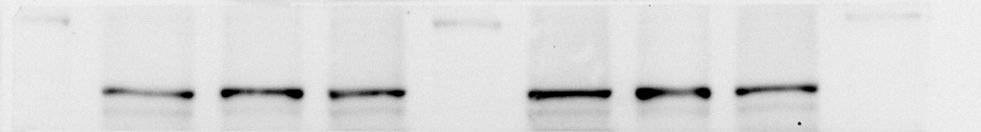

Supplement: Supplemental Information 1 [file peerj-07-7192-s001.zip › western blot/Figure4B NOX1.tif]

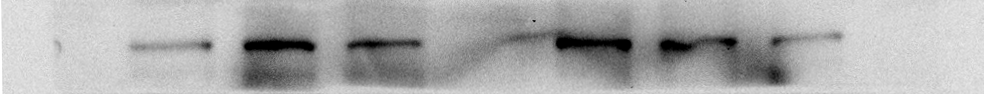

Supplement: Supplemental Information 1 [file peerj-07-7192-s001.zip › western blot/Figure4B NOX2.tif]

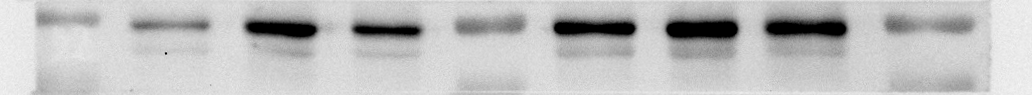

Supplement: Supplemental Information 1 [file peerj-07-7192-s001.zip › western blot/Figure4B NOX4.tif]

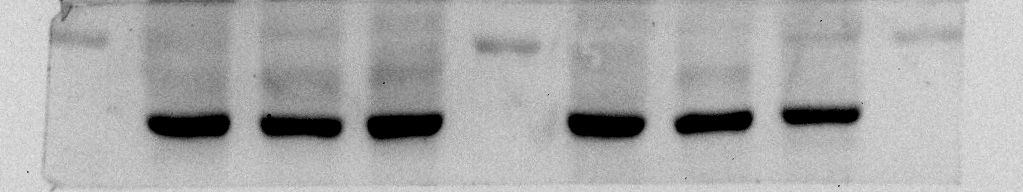

Supplement: Supplemental Information 1 [file peerj-07-7192-s001.zip › western blot/Figure4B a┬-actin.tif]
